# To Our Readers

**Published:** 1896-12

**Authors:** 


### TO OUR READERS.

Many of our readers seem not to have read carefully our repeated notices as to the present business direction of the Journal in relation to unpaid subscriptions. Since the resuscitation of the Journal in September, 1894, we have continued old subscribers on the books for one year, without erasing their names from our subscription list. When that time had expired and the Journals ceased to be sent forth many inquiries were made and surprise expressed because in the past a credit of three, four, or five years was extended. As our printer's bills must be liquidated every thirty days, and as we are expending more for this work than ever before, we consider it unjust to ourselves to extend such long terms of credit, and, accordingly, we have to freshen the memories of our delinquents several times before the bills are paid, all of which is annoying and unpleasant, aside from the added burden of postage, which is no small item of expense in issuing a periodical. For these reasons we have, after twenty-eight months of experience of this plan, decided to abandon the same with this number, which closes Volume XVII. With the beginning of Volume XVIII, January, 1897, we will insist upon all subscriptions being in advance, and our readers will kindly bear this in mind, and should the Journal cease to arrive we would ask of them to look up their last receipt before writing us.

